# Protein ligand binding site prediction using graph transformer neural network

**DOI:** 10.1371/journal.pone.0308425

**Published:** 2024-08-06

**Authors:** Ryuichiro Ishitani, Mizuki Takemoto, Kentaro Tomii

**Affiliations:** 1 Division of Computational Drug Discovery and Design, Medical Research Institute, Tokyo Medical and Dental University, Bunkyo-ku, Tokyo, Japan; 2 Department of Biological Sciences, Graduate School of Science, The University of Tokyo, Bunkyo-ku, Tokyo, Japan; 3 Preferred Networks, Inc., Chiyoda-ku, Tokyo, Japan; 4 Artificial Intelligence Research Center (AIRC), National Institute of Advanced Industrial Science and Technology (AIST), Koto-ku, Tokyo, Japan; Nazarbayev University, KAZAKHSTAN

## Abstract

Ligand binding site prediction is a crucial initial step in structure-based drug discovery. Although several methods have been proposed previously, including those using geometry based and machine learning techniques, their accuracy is considered to be still insufficient. In this study, we introduce an approach that leverages a graph transformer neural network to rank the results of a geometry-based pocket detection method. We also created a larger training dataset compared to the conventionally used sc-PDB and investigated the correlation between the dataset size and prediction performance. Our findings indicate that utilizing a graph transformer-based method alongside a larger training dataset could enhance the performance of ligand binding site prediction.

## Introduction

The identification of the compound binding site of the target protein is the first step in structure-based drug design. In this context, “binding site prediction” is the task of predicting the binding site of its ligand or other compounds on the target protein surface [1–4]. This is especially important when there is no experimental information available about the binding site of its substrate or ligand. This method is also important in the case of targeting sites other than the original substrate binding site. Discovering the allosteric binding site that modulates the activity of the target protein is one of the important tasks in drug discovery [5]. In those cases, binding site prediction is performed first, and then, compounds that bind to the predicted binding sites are designed in a structure-based manner.

Due to the importance of the binding site prediction task, many previous studies have been carried out over several decades, and various methods have been investigated [1]. They can be roughly divided into five categories [6]: geometric [7, 8], energetic [9], conservation-based [10, 11], template-based [12, 13], and machine learning (ML)/knowledge-based methods [6, 14–17]. Recently, ML-based methods have been well-studied. They can be further divided into two types. The first type includes methods that predict a pocket directly from the target protein structure. In that method, they compute descriptor features for points near the target protein or points on the protein surface, and then directly predict whether the points can form a binding site using these features as input to ML. These include P2Rank [6] and DeepSite [14]. The second type includes a hybrid of rule-based and ML-based methods. In them, candidate binding sites are predicted by a rule-based method and then ranked by an ML score to select the true positive sites. This is based on the observation that the binding sites of ligands or small compounds are often located on the concave surface of the protein. It has been shown that the rule-based methods using protein surface geometry can successfully detect the candidates of the positive binding sites with a high recall score [15]. However, as these candidate sites usually include many false positive sites, it is not an easy task to predict true positive sites from them. The program DeepPocket is an example of this type of method [15].

Alternatively, the ML-based methods can be divided into two types, depending on the ML algorithm used, i.e., neural-network (NN) based and non-NN-based methods. The latter includes the program P2Rank (and PRank) that uses the random forest as an ML algorithm. The NN-based methods, especially deep learning (DL)-based methods, have been well studied recently. For example, the programs DeepSite [14], Kalasanty [17], and DeepPocket [15] exploit a 3D convolutional neural network (CNN) to predict the binding sites using a voxelized protein structure as an input.

Recently, methods based on graph convolutional NNs (GCNs) with the property of roto-translation invariance (or equivariance), which can be generalized as transformer-based models [18–22], have successfully been exploited for the protein structure prediction and design tasks [23–25], attracting attention in the field of computational structural biology. In this study, we tried to apply this roto-translation invariant NN to the problem of the binding site prediction. The graph transformer-based model was used in the ML part of the hybrid method, and then its performance was compared with that of previous studies, including 3D CNN-based models. In addition, we measured the effect of the size of the training dataset, by constructing a larger dataset than the sc-PDB [26], which was commonly used in previous studies.

## Methods

### Data preprocessing

To apply the machine learning (ML) methods to the binding site detection problem, there are two possible settings: one is to predict the sites directly from the protein structure, and the other is to predict the candidates using a rule-based method and then rank these candidates by the ML-based method. In this study, we adopted the latter one, i.e., a hybrid of the rule-based and ML methods. For the rule-based method, we used the program Fpocket (version 4.0), which detects the concave surface of protein using the geometry of the protein surface structure and property [7]. As shown by previous study [15], the program Fpocket can predict possible ligand binding sites with high recall scores, i.e., it outputs a binding site that contains the correct answer, but the output contains many false-positive pockets. In this study, we tried to predict true positive pockets with high accuracy from the output of the program Fpocket.

We preprocessed the protein structures to create the input data for the neural network (NN) as follows. First, to calculate possible pockets, Fpocket was run on protein structures after the removal of water and bound ligand molecules, etc. Then, we selected the protein amino acid residues within 10 Å around all pocket vertices (alpha spheres) calculated by Fpocket to construct the pocket residues. The input graph *G* = (*V*, *E*) was constructed from the pocket residues as follows: node *V* = {*v*_*i*_} is defined for each Cα atom *α*_*i*_, where *i* is the index of the pocket residues, and then edge *E* = {*e*_*i*,*j*_} is added between nodes *v*_*i*_ and *v*_*j*_ where the distance between atoms *a*_*i*_ and *a*_*j*_ (*i*≠*j*) is within 25 Å. For the training dataset, grand truth labels were assigned using a commonly used criterion, DCA [27], which is defined as the minimum distance between the pocket barycenter and any ligand atom of the experimental structure. Specifically, the pockets with DCA values less than 4 Å were labeled as true samples, otherwise false samples. Here, it should be noted that all structures in the training dataset were selected to contain more than one bound ligand to ensure that the labels can be assigned using these bound ligands.

### Model

Using the pocket residue data defined above, we constructed a NN model with a hidden dimension of *d* to classify the Fpocket’s prediction as true or false positive pockets. The node feature $f_{i}^{0}$ for node *v*_*i*_ is calculated as follows.

$$f_{i}^{0}=E^{aa}∙\mathrm{aa}\left(v_{i}\right)$$

where **E**^aa^∈ℝ^21,*d*^ is the weight matrix, and aa(∙) is a function to return the one-hot representation ({0,1}^21^) of the amino acid residue of the given node, including naturally occurring 20 amino acids and an unknown token. For the model using the SASA (solvent-accessible surface area) feature of the node, $f_{i}^{0}$ is calculated as follows.

$$S_{i}=\{S_{i}^{k}\}$$

$$S_{i}^{k}=\exp\left(-\gamma {\left(s_{i}-\mu_{k}\right)}^{2}\right)$$

$$f_{i}^{0}=(E^{aa}∙\mathrm{aa}\left(v_{i}\right))⊕S_{i}$$

where *k* = [0, *d*) denotes the index of the feature vector, *s*_*i*_ is the SASA value for the residue corresponding to the node *v*_*i*_. The hyperparameters for the SASA embedding (*γ* and *μ*_*k*_) was determined so that the centers of the radial basis function are equally located in the range of 0 Å^2^ and 350 Å^2^. The upper limit of the SASA embedding (350 Å^2^) was determined based on the SASA distribution of the amino acid residues in the dataset (S1 Fig). The edge feature $e_{ij}=\{e_{ij}^{k}\}$ is calculated as follows.

$$e_{ij}^{k}=\exp\left(-\gamma {\left(\|r_{i}-r_{j}\|-\mu_{k}\right)}^{2}\right)$$

where **r**_*i*_ is the Cartesian coordinates of the atom corresponding to the node *v*_*i*_. The hyperparameters for the distance embedding (*γ* and *μ*_*k*_) was determined so that the centers of the radial basis function are equally located in the range of 3–25 Å.

**Fig 1:**
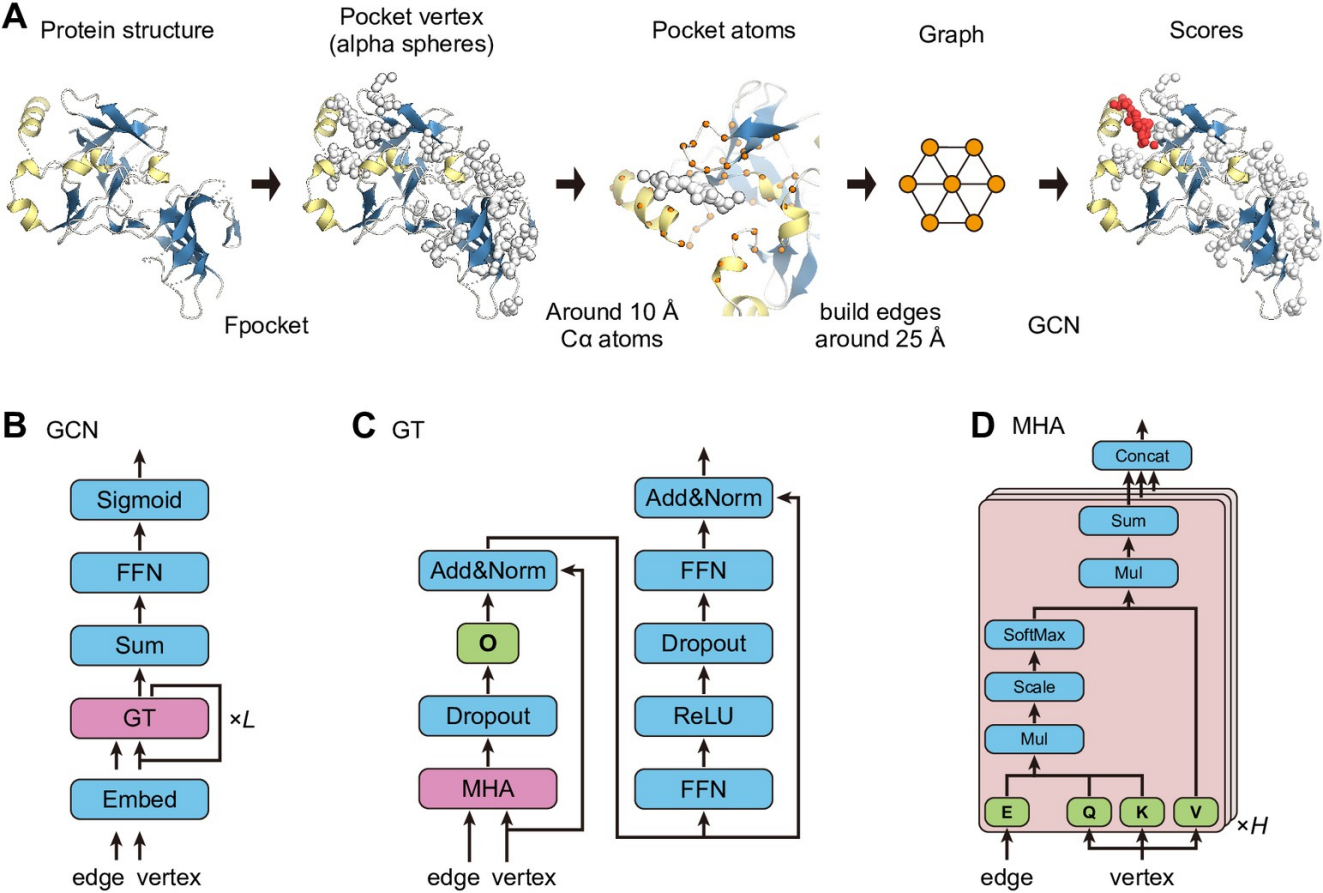
The ligand binding site prediction model proposed in this paper. A) Schematic diagram of the procedure for the prediction. A pocket vertex is calculated by processing the input protein structure with Fpocket. The graph is generated from Cα atoms within 10 Å around the pocket vertex, which is then used as input to NN to calculate the ligand-binding site score. B, C, D) Schematic diagrams of the graph transformer neural network. Green boxes represent weight tensors for query (Q), key (K), value (V), edge (E), and output (O). Magenta boxes represent graph transformer (GT) and multi-head attention (MHA) modules, respectively. The details of GT and MHA modules are depicted in panels (C) and (D), respectively.

Using these input feature vectors ($f_{i}^{0}$ and e_*ij*_), the node feature vectors ($f_{i}^{l}$ where *l* = [0, *L*)) were updated using the graph transformer [28] with a hidden dimension of *d* defined as follows (Fig 1).

The query, key, and value projections $q_{i}^{h},k_{i}^{h},v_{i}^{h}$ of the node feature vectors $f_{i}^{l}$ were calculated using the weight matrices $Q^{h,l},K^{h,l},V^{h,l}\in R^{d^{h},d}$, respectively, as in the original transformer paper [29] (Fig 1C). Note that *d*^*h*^ is the dimension of a head satisfying the following relation *d*^*h*^∙*H* = *d*, where *H* is the number of heads.


$$q_{i}^{h},k_{i}^{h},v_{i}^{h}=\mathrm{Linear}\left(f_{i}^{l}\right)$$

The edge projection $b_{ij}^{h}$ of the edge feature vector **e**_*ij*_ was also calculated using the weight matrix $E^{h,l}\in R^{d^{h},d}$ (Fig 1C).


$$b_{ij}^{h}=\mathrm{Linear}\left(e_{ij}\right)$$

Using these key, query, and edge projections ($q_{i}^{h},k_{i}^{h},e_{i}^{h}$) the attention $a_{ij}^{h}$ was calculated as follows.


$$a_{ij}^{h}={\mathrm{softmax}}_{j}\left(\frac{1}{\sqrt{d^{h}}}\sum_{k}q_{ik}^{h}∙k_{jk}^{h}∙b_{ijk}^{h}\right)$$

The output of the above softmax function was clamped between –5 and 5 for numerical stability. The attention was calculated only for the node pairs (*i*, *j*) directly connected by the edges. Then, the output of the multi-head attention $o_{i}^{h}$ was calculated using the value projections and the attentions calculated above.


$$o_{i}^{h}=\sum_{j}a_{ij}^{h}v_{j}^{h}$$

The outputs of the heads were concatenated and passed to the feed forward network and normalization layers including skip connections to calculate the final output of the graph transformer layer $f_{i}^{l+1}$:

$${\tilde{f}}_{i}^{l}=\mathrm{Norm}\left(\mathrm{Linear}\left(\mathrm{Drop}\left({\mathrm{concat}}_{h}\left(\left\{o_{i}^{h}\right\}\right)\right)\right)+f_{i}^{l}\right)$$

$$f_{i}^{l+1}=\mathrm{Norm}\left(\mathrm{Linear}\left(\mathrm{Drop}\left(\alpha (\mathrm{Linear}\left({\tilde{f}}_{i}^{l}\right))\right)\right)+{\tilde{f}}_{i}^{l}\right)$$

where *h* = [0, *H*) denotes the index of heads, Linear(∙) a fully connected linear layer, softmax_*j*_(∙) the softmax function over the index *j*, concat_*h*_(∙) concatenation of the vectors over the index *h*, Drop(∙) a drop-out layer [30], *α*(∙) an activation function, and Norm(∙) a layer-wise normalization layer [31]. We used the rectified linear unit (ReLU) as the activation function, i.e., *α*(*x*) = max(*x*, 0). While the node features ($f_{i}^{l}$) were updated by each layer as above, the edge features were not updated, since almost no performance gain was observed. The resulting graph transformer layers were stacked *L* times to calculate the final feature vector $f_{i}^{L}$ (Fig 1B). Then, the hidden vector **h** for the pocket graph *G* was calculated using the pooling of the hidden vectors $f_{i}^{L}$. We tried pooling methods suggested in previous studies [28, 32, 33] and found that sum pooling yielded the best results in our current study.


$$h=\sum_{i}f_{i}^{L}$$

Finally, the loss function ℒ for the binary classification task is calculated as follows.

y=σ(ϕ(h))

$$L=-y^{*}\log y-(1-y^{*})\log\left(1-y\right)$$

where *ϕ* is a multi-layer perceptron (MLP) with ReLU activation function, *σ* is a sigmoid function, and y*∈{0,1} is a ground truth label of the corresponding pocket. Overall, the loss function ℒ satisfies the E(3) invariance according to the translation and rotation of the input atom coordinates.

### Training

The loss function ℒ defined above was minimized for the training dataset using the Adam optimizer [34]. Cosine annealing with a warm-up with a period of 25 epochs and a maximum learning rate of 2×10^−4^ was used to schedule the learning rate of the optimizer. The batch size was 128, and 300 epochs of training were performed in total. The model with the highest PR-AUC (area under the precision-recall curve) value for the validation dataset was saved as the best model during the training.

### Data augmentation

Although the candidate pockets generated by the program Fpocket contain true positives, the majority of them are false positives, thereby making the dataset highly imbalanced. Therefore, if the dataset is used as is for training, it is difficult to obtain models with high recall and precision. To mitigate this label imbalance and obtain a model with good performance, the true-label samples were augmented to a level equal to the number of false-label samples. However, simply repeating the true-label samples in the training process could result in overfitting. In this study, we tried to suppress this overfitting problem by adding noise to the training data. More specifically, we tried to prevent overfitting by adding noise to the dataset in the following ways:

1. Adding normal distributed noise to the Cartesian coordinate values of atom positions (**r**_*i*_) according to the following formula.
$$r_{i}≔r_{i}+\sigma_{pos}∙\epsilon \;(positional\;noise)$$
  where ϵ∼N(0,1) and *σ*_pos_ is a hyperparameter that controls the strength of the positional noise.
2. Randomly dropping/duplicating nodes by sampling (1−*σ*_node_)∙*N*_orig_ nodes with replacement from the original *N*_orig_ nodes in the dataset (node dropping), where ϵ∼N(0,1) and *σ*_node_ is a hyperparameter that controls the node dropping.
3. Adding normal distribution noise to the SASA values according to the following formula.
$$s_{i}≔s_{i}∙(1+\sigma_{SASA}∙\epsilon )\;(SASA\;noise)$$
  where *σ*_SASA_ is a hyperparameter that controls the strength of the SASA noise.

### Datasets

In this study, we created two datasets of different sizes to compare performance along with the model size. The first one is a dataset based on the sc-PDB v.2017 database [26] containing 16,247 PDB entries. Next, a test set was created to evaluate the model performance. We here used a union of the coach420 and holo4k datasets [6] as the test set, which were commonly used in previous studies [6, 15]. To prevent leakage between the training/validation and test sets, amino acid sequences with more than 50% sequence identity to those of the test set proteins were excluded from the training/validation set (per previous studies [15]). As a result, 7,710 PDB entries were removed from the original dataset, leaving 8,537 PDB entries in the training/validation set. This dataset was processed by the program Fpocket [7], resulting in 276,531 pocket candidates with about 7.1% positives. The samples were randomly split into five parts and a 5-fold cross-validation was performed.

The second is a dataset based on the PoSSuM database [35]. Among the known ligand-binding sites deposited in PoSSuM database, we extracted the entries that bind ligands that appeared in the sc-PDB dataset. As a result, the dataset contains 37,067 PDB entries. As in the case of the sc-PDB dataset, we used a union of coach420 and holo4k dataset as the test set [6]. The entries with sequence identity greater than 50% against the test set proteins were removed from the training/validation set. As a result, 22,599 PDB entries remained. This dataset was processed using Fpocket, resulting in 729,853 pocket candidates with about 6.3% positives. The samples were randomly split into five parts and a 5-fold cross-validation was performed.

## Results

To evaluate the performance on imbalanced datasets, we compared the PR-AUC value as the evaluation metric. We also compared ROC-AUC and “Top-(*n* + *i*) success rate” to compare with previous methods. The definition of the Top-(*n* + *i*) success rate followed the previous study [15]. For the evaluation of the overall performance, we calculated above metrics for the prediction results on the test set (i.e., coach420 and/or holo4k dataset) to avoid leakage from the training dataset. The ensemble average of the outputs from the five models by the 5-fold cross-validation was used as the prediction.

**Fig 2:**
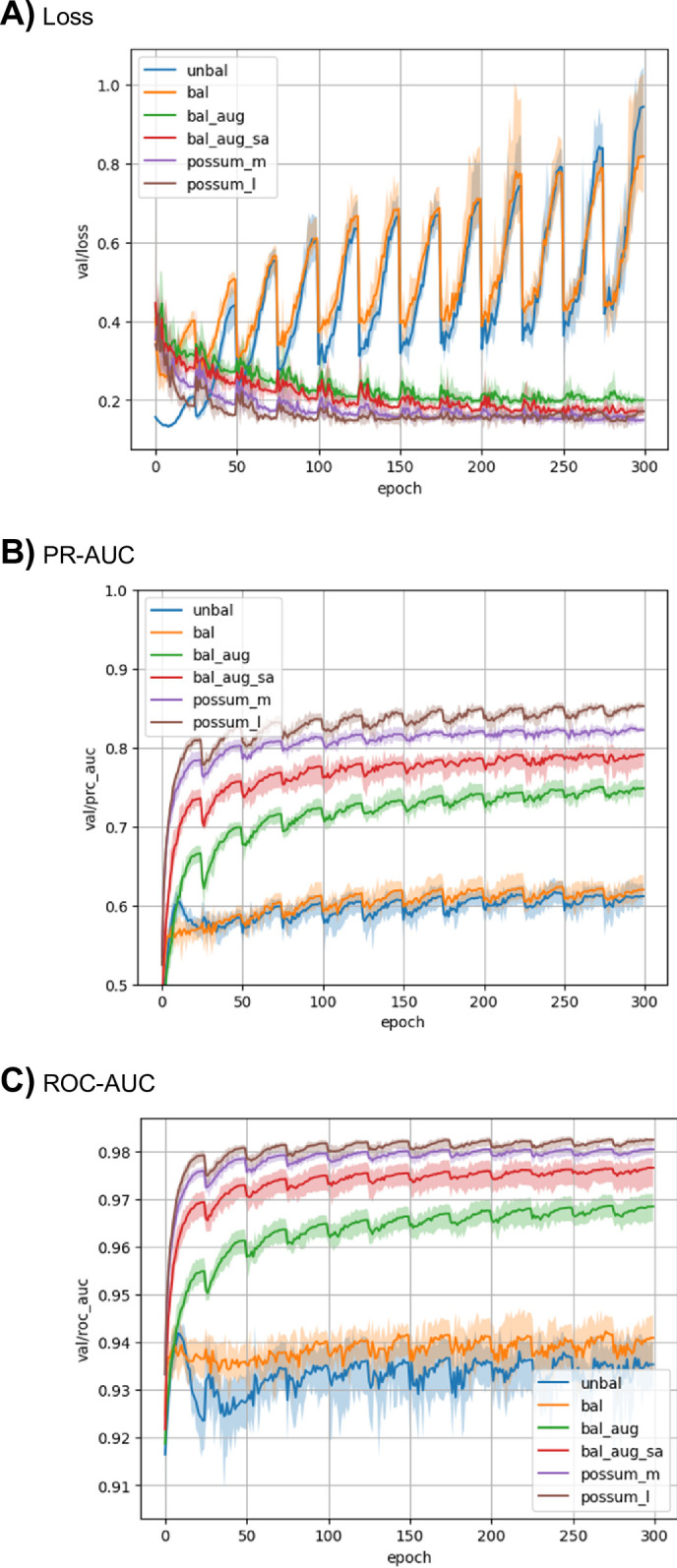
The learning curves for the models trained in this study. A) Loss, B) PR-AUC, and C) ROC-AUC values for the validation dataset were plotted. The mean of the 5 models resulting from the cross-validation is plotted as a solid line, while the values for each model as a transparent line. Please refer to Table 1 for the abbreviations used in the figure legends.

**Fig 3:**
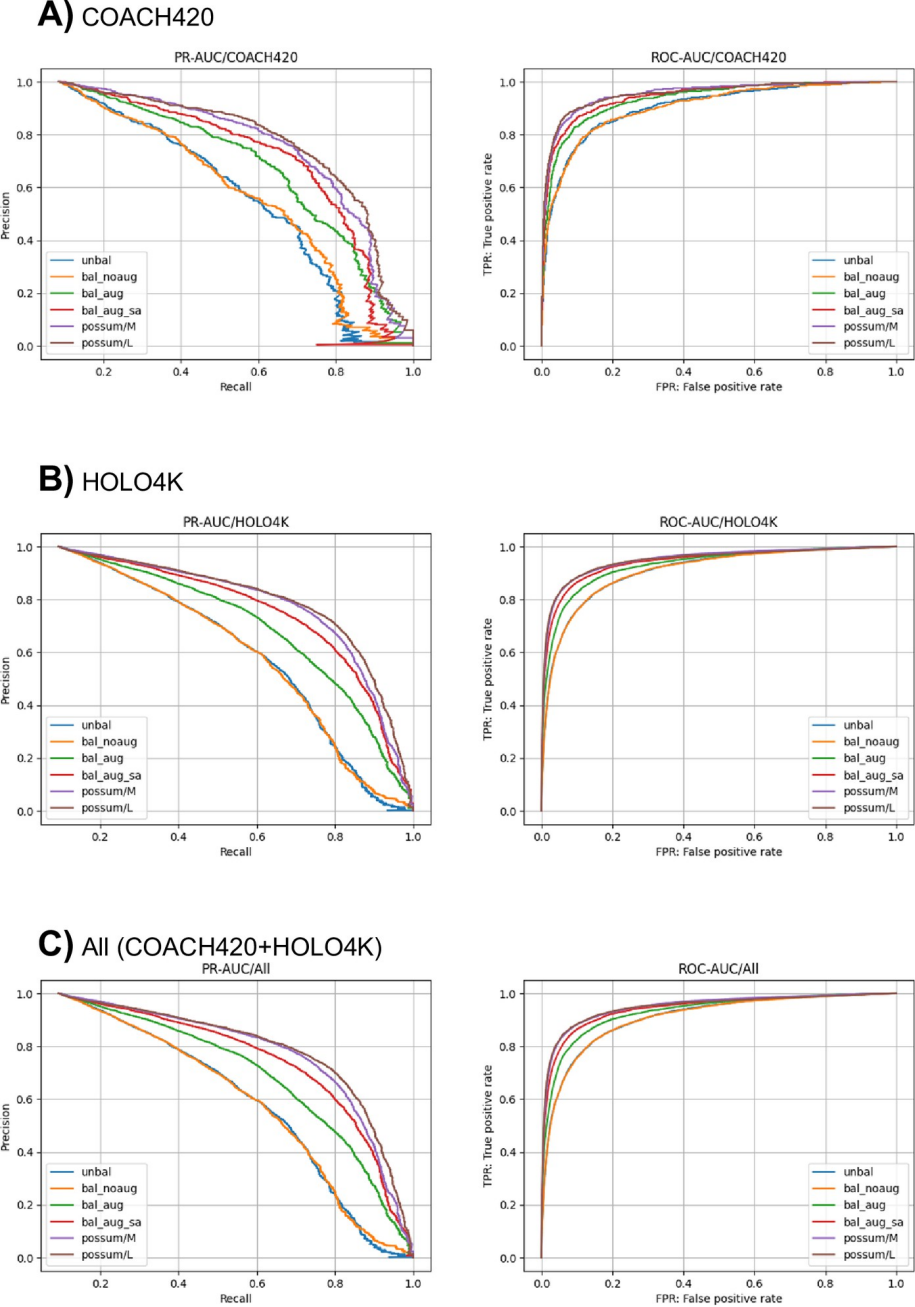
Plots of precision-recall (PR) and receiver-operating-characteristic (ROC) curves against the test dataset (A: coach420, B: holo4k, and C: holo4k+coach420).

At first, we trained the model with baseline hyperparameters (Table 1) on the sc-PDB dataset and evaluated its performance. The resulting model size is ~1.28 M parameters. As expected, the case without data balancing on the training dataset, i.e., using the dataset as is for training (Unbal; Table 1), resulted in a model with poor performance. In particular, the loss function on the validation set only decreased for the first few epochs, and after that, it increased significantly (Fig 2A). Although ROC-AUC exhibited a high value of about 0.93, the PR-AUC value reached a plateau at around 0.6 (Fig 2B), indicating that the model’s performance on predicting positive binding sites is not high. Next, we evaluated the performance of the trained model against the test dataset. Like the performance against the validation set, the PR-AUC value is not high, indicating poor performance on the positive binding sites prediction (Table 2, Fig 3).

**Table 1:** Summary of the experiments performed in this work.

	Balancing	Noise	SASA	*L*	*d*	Dataset
Unbal	off	off	off	8	128	sc-PDB
Bal	on	off	off	8	128	sc-PDB
Bal+aug	on	on	off	8	128	sc-PDB
Bal+aug+SA	on	on	on	8	128	sc-PDB
PoSSuM/M	on	on	on	8	128	PoSSuM
PoSSuM/L	on	on	on	12	256	PoSSuM

**Table 2:** Summary of the PR-AUC and ROC-AUC metrics for the test datasets.

	PR-AUC			ROC-AUC		
	coach420	holo4K	All	coach420	holo4K	All
Unbal	0.5808	0.6172	0.6127	0.9036	0.9095	0.9086
Bal	0.5915	0.6176	0.6139	0.9028	0.9079	0.9070
Bal+aug	0.6907	0.7158	0.7115	0.9342	0.9305	0.9302
Bal+aug+SA	0.7236	0.7691	0.7640	0.9450	0.9424	0.9423
PoSSuM/M	0.7632	0.7933	0.7896	**0.9543**	**0.9500**	**0.9500**
PoSSuM/L	**0.7816**	**0.8067**	**0.8035**	0.9531	0.9490	0.9489

**Fig 4:**
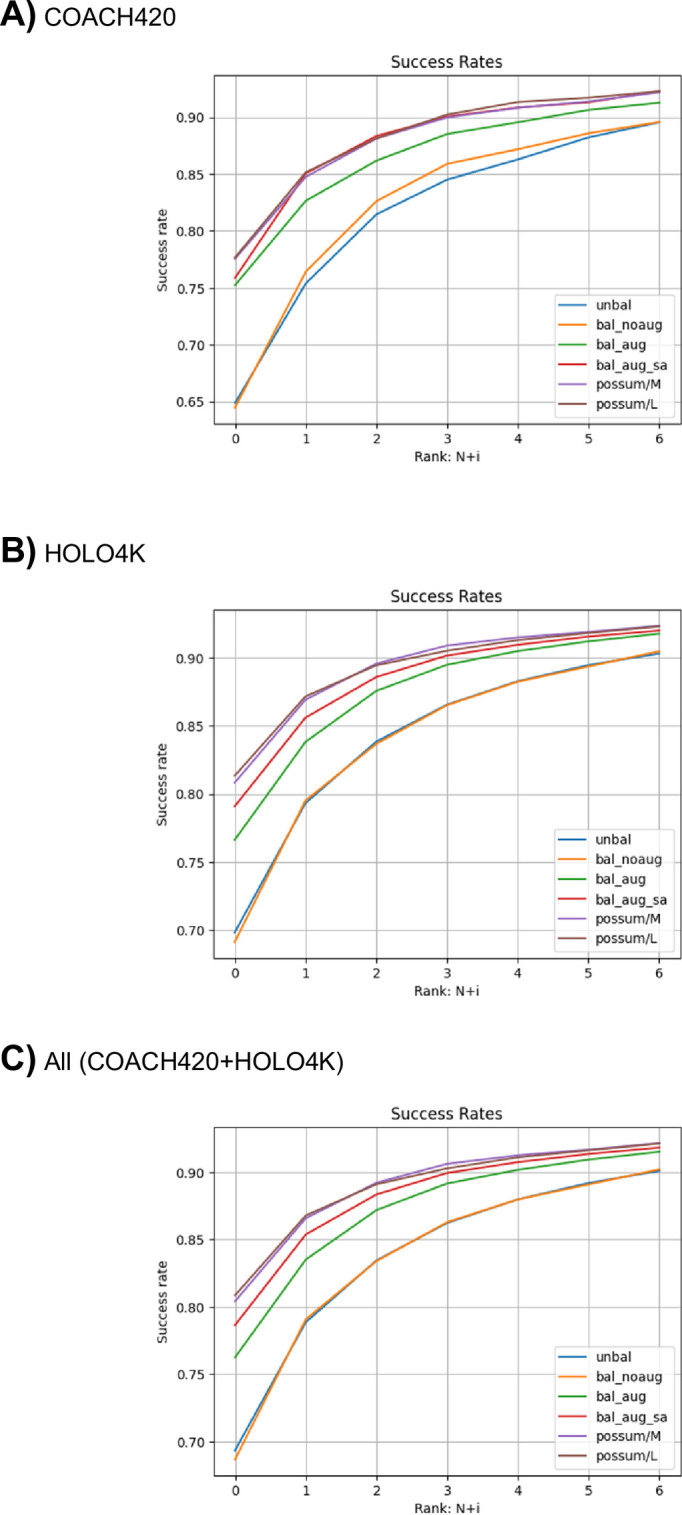
Plots of success rates (*i* = 0 ~ 6) against the test dataset (A: coach420, B: holo4k, and C: holo4k+coach420).

Next, the model with the same hyperparameters was trained with label balancing (Bal; Table 1). The results showed that the PR-AUC values for the validation dataset improved slightly during the training epochs, and the best PR-AUC value is slightly improved (~0.62) as compared to the case without balancing (Fig 2B). However, similar to the case without balancing, the loss function for the validation set tends to increase after the first few epochs (Fig 2A). The inference result for the test dataset showed almost the same performance as that without label balancing in terms of PR-AUC and success rate (Figs 3 and 4). The negative effect of overfitting to the same true-positive samples may be stronger than the effect of label balancing.

Next, the model with the same hyperparameters was trained including positional noise addition and node dropping, in addition to the label balancing (Bal+aug; Table 1). The tuning of the hyperparameters controlling the noise amounts (*σ*_pos_ and *σ*_node_) were performed and we found that *σ*_pos_ = 0.5 and *σ*_node_ = 0.03 gives the best result. The loss function for the validation set gradually decreased over 300 epochs (Fig 2A), and the PR-AUC value increased over 0.75. Accordingly, the PR-AUC value against the test dataset significantly increased (Fig 3, Table 2). The success rate also showed improvements as compared to those of other NN-based methods (Table 3). The data augmentation by noise addition may be effective in suppressing overfitting to the true-positive samples.

**Table 3:** Summary of the success rates for the test dataset including the results from previous studies. The success rate values of the previous studies are taken from ref. [15].

	Coach420		Holo4k	
	Top-n	Top-(n+2)	Top-n	Top-(n+2)
Fpocket	35.09%	51.25%	36.34%	51.53%
DeepSite	53.07%	53.07%	51.65%	51.67%
Kalasanty	63.51%	65.18%	61.21%	62.63%
P2Rank	68.24%	75.48%	70.6%	80.05%
DeepPocket	67.96%	79.94%	73.36%	82.97%
PoSSuM/L	**77.67%**	**88.14%**	**81.33%**	**89.43%**

In addition, the SASA features were added to the training dataset (Bal+aug+SA; Table 1). Noise was also added to the SASA features as described in the Methods section. The tuning of the noise amount for the SASA values were also performed, and we found that *σ*_SASA_ = 0.3 gives the best result. The training results showed that the PR-AUC value for the validation dataset was significantly improved and reached 0.8 (Fig 2B). The result of the test dataset inference also showed that the model performance improved in both PR-AUC and success rate (Figs 3 and 4, Table 2). These results seem to be reasonable because previous studies have shown that the SASA is an important feature for the ligand binding site prediction [6].

Next, the PoSSuM dataset, which is about 2.6 times larger dataset than the sc-PDB dataset, was used to train in the same conditions, including the model size, label balancing, noise addition, and SASA features. (PoSSuM/M; Table 1). As a result, improvement was observed in both PR-AUC values to the validation and test dataset (Figs 2B and 3) as well as success rate (Fig 4). We also trained a larger model with ~7.34 M parameters using the PoSSuM dataset. (PoSSuM/L; Table 1). A slight improvement in PR-AUC for validation and test datasets was observed (Figs 2B and 3); however, the increase in the success rate is limited to a marginal level (Fig 4). The capacity of the baseline model is likely sufficiently large even for the PoSSuM dataset, and thus increasing the model size did not significantly improve performance.

**Fig 5:**
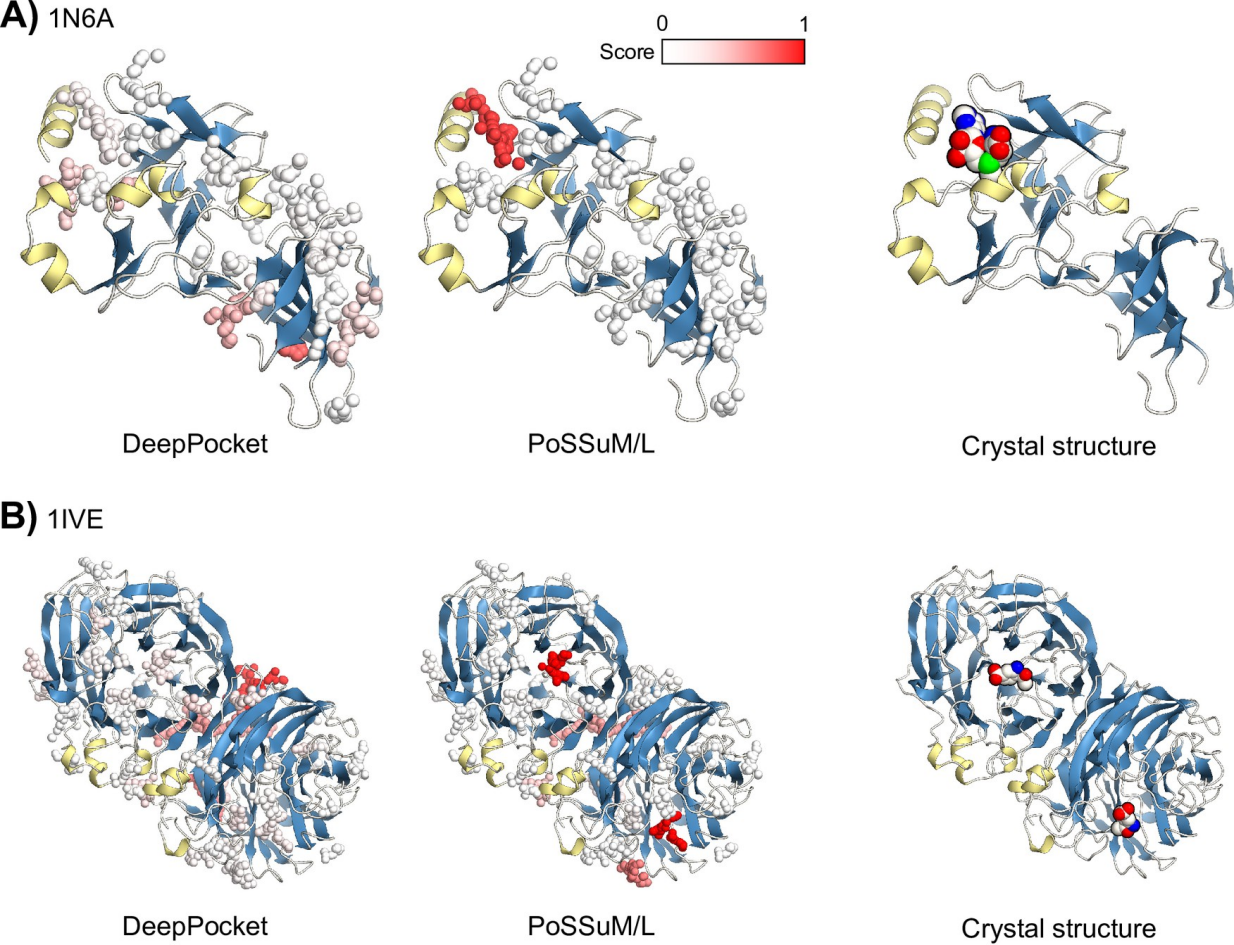
Examples of the ligand binding site prediction. The prediction results for the protein structures of (A) SET7/9 lysine methyltransferase (PDB ID: 1N6A) and (B) influenza virus neuraminidase (PDB ID: 1IVE) in the test dataset by the model in this study (PoSSuM/L) and DeepPocket [15] are shown in the left and center panels, respectively. The pocket vertices calculated by Fpocket were shown by spheres colored from white to red according to the output values (0–1) of the NN model. The actual positions of the ligand in the crystal structures were shown in the right panels.

Finally, when compared in terms of success rate, the best model performed better than the previous methods, including other NN-based methods such as DeepPocket (Table 3). In contrast, the ROC-AUC values (Table 2) do not improve significantly compared to DeepPocket (0.951 for holo4k); however, it should be considered that the ROC-AUC value itself is not an appropriate criterion to evaluate models trained using an imbalanced dataset. The examples of the prediction by the best model (PoSSuM/L) and DeepPocket are shown in Fig 5. In these examples, DeepPocket failed to predict the correct site, while PoSSuM/L successfully predicted the correct site with high contrast.

## Discussion

In this study, we built a model for ligand binding site prediction using a combination of the rule-based method and the graph transformer-based NN model. We found that data augmentation of the graph structure, including the addition of noise to the atom positions and the random removal and/or addition of graph nodes, is crucial to avoid overfitting to the highly imbalanced training datasets. We also created a dataset based on the PoSSuM database [35] and examined the effect of increasing model size and dataset size. The best model showed improved performance in terms of success rate compared to methods in previous literature, including other NN-based and rule-based methods (Table 3).

The GCNs, including the graph transformer used in this study, have several advantages over the 3D-CNN used in previous research. First, 3D-CNNs have several hyperparameters for input features, such as the extent of voxelization region and voxel resolution, while GCNs do not have such hyperparameters since they can directly input protein atoms as point clouds. Furthermore, the use of an *E*(3)- or *SE*(3)-invariant GCN eliminates the need for augmentation of the rotation and translation of the input data, thereby allowing an efficient training process. Second, GCNs can utilize a variety of atom and residue features, including discrete and continuous values, as node properties. For example, the use of high-dimensional output vectors of the protein language models [36] as the node feature of specific amino acid residues would be even possible. In the 3D CNN-based models, input features (e.g., atom types) are encoded as different channels, and thus it would be difficult to efficiently use continuous or multi-dimensional values as input features. In contrast, there are also advantages of the 3D-CNN-based models. For example, DeepPocket, which is one of the 3D-CNN-based methods, contains a model to predict the shape of the binding sites [15]. In this method, the shape of the binding site is predicted by segmentation of volume data using the U-Net-like model [37]. Although recent works applied GCN-based models to the image segmentation tasks [38], it may not be straightforward and performance-effective to apply the GCN-based models for the 3D voxel segmentation around the binding sites.

Concerning the dataset size, larger datasets were shown to contribute to better prediction performance in the current problem setting (Fig 3). The available databases such as sc-PDB [26] and PDBBind [39] only include curated PDB entries. The inclusion of a wide range of uncurated PDB entries that are bound to small molecules may enable the construction of larger datasets and contribute to improved prediction performance. Recently, the latest version of PoSSuM provides a pocket database that includes the predicted structures [40]. Data augmentation through the addition of such non-experimental structures may further improve prediction performance.

## Supporting information

S1 Fig (PDF)
