# Supplementary material for: Protein ligand binding site prediction using graph transformer neural network
Source: PLoS One. 2024 Aug 6;19(8):e0308425. doi: 10.1371/journal.pone.0308425 (PMC11302905; doi:10.1371/journal.pone.0308425)
Supplement: S1 Fig — (PDF) [file pone.0308425.s001.pdf]

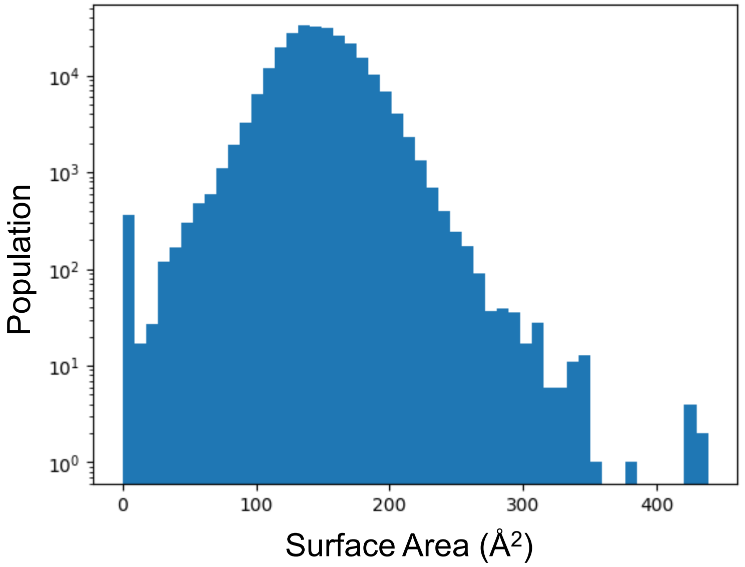

SI Fig. 1

Distribution of solvent contact surface area per amino acid residue. The solvent contact surface area (Å^2) is shown on the horizontal axis, while the number of residues is displayed on the vertical axis, which is set on a logarithmic scale.
